# Single screen of citations with excluded terms: an approach to citation screening in systematic reviews

**DOI:** 10.1186/s13643-018-0782-x

**Published:** 2018-07-28

**Authors:** Brittany U. Carter

**Affiliations:** Kaiser Permanente Care Management Institute, 1 Kaiser Plaza, Oakland, CA USA

**Keywords:** Systematic review, Methodology, Literature search, Exclusion criteria, Abstract screening

## Abstract

Citation screening during the systematic review process can be time-consuming due to the exponentially increasing amount of research. This letter describes an approach to expediting the process by single screening citations that include terms in the abstract and/or keywords related to the exclusion criteria of the systematic review to quickly reject studies with a high likelihood of being excluded from the systematic review. This method can potentially improve the efficiency of the citation screening process while maintaining the quality of the systematic review; however, future research is needed to further validate this approach.

## Background

Systematic reviews aim to identify all empirical evidence to answer a specific research question [1]. As part of the systematic review process, comprehensive literature searches of multiple databases are performed to identify all potentially relevant studies. The number of citations identified for a systematic review varies greatly based on the complexity of the systematic review and research questions (e.g., number of questions, inclusion criteria), the number of literature databases searched, the search strategies employed, and the range of search dates used. For some systematic reviews, thousands (or tens of thousands) of citations of potentially relevant studies are identified [2, 3]. As a result, systematic reviewers are exploring methods to improve the accuracy and efficiency of the citation screening process without compromising the quality of the systematic review [4–9]. This article describes an approach to expediting the citation screening process by single screening citations that include terms in the abstract and/or keywords related to the exclusion criteria of the systematic review to quickly reject studies that have a high likelihood of being excluded from the systematic review.

## Approach

An initial literature search designed by a research librarian was conducted for a U.S. Preventive Services Task Force systematic review on the use of non-traditional risk factors in cardiovascular disease risk assessment [10]. The initial search yielded 18,360 unique citations; citations were managed in EndNote™ version 7.3.1 (Thomson Reuters, New York, N.Y.). The final research questions, protocol, and systematic evidence review [10], including the literature search strategies and study selection criteria, can be found at https://www.uspreventiveservicestaskforce.org.

After receiving the results of the initial search, but prior to initiating citation screening, a list of 83 terms (50 truncated terms) related to an excluded setting, population, or condition was developed based on the exclusion criteria of the systematic review (Table 1); from here on, these terms are labeled as “excluded terms.” In EndNote, these excluded terms were searched for in the abstract and/or keyword fields (using an asterisk for truncation) in groups of 10 (the maximum allowable number of terms in a search group) using the Boolean operator “OR.” Due to the limited number of groups of terms that can be combined into a “group of groups” (10 groups), one group of 10 excluded terms was only searched for in the abstract field. The nine groups of excluded terms were batched together using the Boolean operator “OR” and then separated from the group of citations that did not have these excluded terms in their abstract and/or keywords using the Boolean operator of “NOT” (reaching the limit of 10 groups in a “group of groups”). These citations were single-screened by the author for relevancy using the pre-specified inclusion and exclusion criteria of the systematic review, with single citations being either rejected or moved forward for dual abstract screening. Investigators screened citations in DistillerSR (Evidence Partners, Ottawa, Canada), an online-based systematic review software program.

**Table 1:** List of excluded terms

Exclusion criteria	Excluded terms^d^	Truncated excluded term^a^	No. of citations with term in abstract^b^	No. of citations with term in keywords^b^
Condition: • Studies that exclusively include individuals with other pre-existing health conditions (e.g., HIV)	Arthritis, arthritic	Arthrit^a^	267	183
	Autoimmune	Autoimmun^a^	93	21
	Cancer	Cancer^a^	307	18
	Hepatitis, hepatic	Hepati^a^	162	24
	HIV	HIV^a^	197	135
	Infection, infectious	Infect^a^	736	349
	Kidney	Kidney^a^	813	789
	Lupus	Lupus^a^	127	112
	Pancreatic, pancreas	Pancrea^a^	35	25
	Renal	Renal^a^	1135	781
	Rheumatology, rheumatic	Rheum^a^	336	223
	Sickle cell anemia	Sickle^a^	9	6
	Transplant, transplantation	Transplant^a^	281	255
	Prader-Willi syndrome	Wille^a^	181	92
Country: • Studies not conducted in countries categorized as “Very High” on the 2014 Human Development Index (as defined by the United Nations Development Program)	Brazil, Brazilian	Braz^a^	64	65
	China, Chinese	Chin^a^	621	249
	Cuba, Cuban	Cuba^a^	51	1
	Egypt, Egyptian	Egypt^a^	17	23
	Haiti, Haitian	Haiti^a^	1	0
	India, Indian	India^a^	136	90
	Iran, Iranian	Iran^a^	53	28
	Iraq, Iraqi	Iraq^a^	3	1
	Kenya, Kenyan	Keny^a^	0	0
	Sri Lanka, Sri Lankan	Lanka^a^	3	1
	Libya, Libyan	Libya^a^	0	0
	Mexico, Mexican	Mexic^a^	33	25
	Nigeria, Niger, Nigerien, Nigerian	Niger^a^	8	10
	Peru, Peruvian	Peru^a^	42	26
	Russia, Russian	Russ^a^	37	21
	Sub-Sahara, sub-Saharan	Sahara^a^	11	1
	South Africa, South African	South Africa^a^	23	19
	Turkey, Turkish	Turk^a^	37	95
	Ukraine, Ukrainian	Ukrain^a^	1	0
Non-human: • Animal studies	Animal	Animal^a^	221	--^c^
	Bovine	Bovin^a^	6	--^c^
	Canine	Canin^a^	9	--^c^
	Feline	Felin^a^	1	--^c^
	Mammal, mammalian	Mammal^a^	11	--^c^
	Mice	Mice^a^	122	109
	Mouse	Mouse^a^	49	--^c^
	Murine	Murine^a^	17	--^c^
	Pig	Pig^a^	50	--^c^
	Primate	Primat^a^	4	0
	Sprague-Dawley rat	Sprag^a^	15	--^c^
	Swine	Swin^a^	8	--^c^
Population: • Children age < 8 years • Pregnant women	Adolescent, adolescence	Adolesc^a^	227	805
	Child, children	Child^a^	565	524
	Infant, infantile	Infant^a^	73	177
	Neonate, neonatal	Neonat^a^	46	9
	Pregnant, pregnancy	Preg^a^	253	134
Total	83 terms	50 truncated terms	7497	5426

^a^Asterisk indicates truncation of search term

^b^Citation counts are not mutually exclusive

^c^Due to limited number of terms and groups of terms in EndNote, these terms were only searched for in the abstract field

^d^Possible excluded terms based on truncation

A total of 6503 (35.4%) of the 18,360 citations from the initial search contained excluded terms in the abstract and/or keywords; these citations were screened by a single reviewer. The remaining 11,847 citations underwent traditional dual screening. Only 246 (3.8%) of 6503 citations containing excluded terms were moved forward for dual screening as they were deemed potentially relevant for inclusion, of which 39 (0.6% of 6503 citations with excluded terms) underwent dual full-text review. Among 39 articles reviewed at full-text, only 2 (0.03% of the 6503 citations with excluded terms) were included in the systematic review. The remaining 37 articles were excluded due to reporting on the wrong outcomes, being the wrong study design, or evaluating the wrong risk prediction base model. The two citations included in the systematic review did not influence the conclusions of the review. None of the 6257 citations with excluded terms—which did not undergo a second review—that were immediately rejected at single screening were brought forward for dual screening or full-text review based on checking the reference lists of included studies or existing systematic reviews. In total, 99.9% of the 6503 citations with excluded terms were excluded from the systematic review. Although these results only describe the initial search of the systematic review, these percentages are likely to not change with the inclusion of citations identified from bridge searches or after expert review and public comment.

## Discussion

Single screening of citations with terms in the abstract and/or keywords that are related to the exclusion criteria of the systematic review is a potential method to increase the efficiency of the citation screening process as it reduces the number of citations requiring dual screening. It also has the potential to be reliable method as 99.9% of the 6503 citations with excluded terms were excluded from the systematic review. This approach would need to be reproduced in other systematic reviews to determine if it is a valid, reliable, accurate, and efficient method to citation screening. Single screening of citations with excluded terms was appropriate for this topic given its high literature yield and the a priori knowledge that most of the citations would not be relevant. This approach, however, may only work well (or be necessary) in topics that have a high literature yield and not those that have less restrictive inclusion criteria (e.g., any country). The amount of time spent on single and dual screening would also need to be compared to truly evaluate efficiency, which was not captured in this study. An optimal method for identifying and batching excluded terms is also needed. In this study, the selection of excluded terms was not systematic and could have been better informed by screening a few hundred citations to identify excluded terms that were more frequent or to omit those that might lead to false excludes. For example, “Kenya, Kenyan” and “Libya, Libyan” yielded zero citations, while “stent” (not an excluded term used) yielded 1480 citations. More excluded terms could also have been used if EndNote was not used to search for citations with excluded terms, as EndNote’s search function limited the number of terms to be searched and grouped. Excluded terms could be integrated into the original literature search strategies and imported separately into EndNote from those that did not have an excluded term in the abstract and/or keywords. And finally, an evaluation of whether the precautions to ensure all relevant studies are identified (i.e., examining reference lists of included studies and existing systematic reviews) is needed to determine that eligible studies with excluded terms were not wrongfully excluded during the single screen. There is still a possibility—even with reference mining—of a false negative as a result of excluding a relevant citation published since included studies or systematic reviews were completed.

Citations identified for a systematic review should at least be single-screened for eligibility, and citations with an excluded term should not be automatically rejected as the excluded term might be in the introduction or background section of the abstract. Due diligence should be taken to ensure no studies were wrongfully excluded by the single screener such as examining references lists of included studies and existing systematic reviews and querying experts for relevant citations. While more efficient processes are being developed and tested, such as machine learning, single screening citations that have a high likelihood of being excluded based on terms in the abstract and/or keywords related to the exclusion criteria of the systematic review can help reduce the number of citations requiring dual screening and thus improve efficiency while maintaining the quality of the systematic review. Additional research is needed to validate this approach.

## References

[1] Higgins JPT, Green S. Cochrane handbook for systematic reviews of interventions. West Sussex: Wiley; 2008.

[2] Bastian H, Glasziou P, Chalmers I (2010). Seventy-five trials and eleven systematic reviews a day: how will we ever keep up?. PLoS Med.

[3] Mallett S, Clarke M (2002). The typical Cochrane review. How many trials? How many participants?. Int J Technol Assess Health Care.

[4] Edwards P, Clarke M, DiGuiseppi C, Pratap S, Roberts I, Wentz R (2002). Identification of randomized controlled trials in systematic reviews: accuracy and reliability of screening records. Stat Med.

[5] Hempel S, Shetty KD, Shekelle PG, Rubenstein LV, Danz MS, Johnsen B, Dalal SR (2012). Machine learning methods in systematic reviews: identifying quality improvement intervention evaluations.

[6] Mateen FJ, Oh J, Tergas AI, Bhayani NH, Kamdar BB (2013). Titles versus titles and abstracts for initial screening of articles for systematic reviews. Clin Epidemiol.

[7] Ng L, Pitt V, Huckvale K, Clavisi O, Turner T, Gruen R, Elliott JH (2014). Title and Abstract Screening and Evaluation in Systematic Reviews (TASER): a pilot randomised controlled trial of title and abstract screening by medical students. Syst Rev.

[8] Rathbone J, Hoffmann T, Glasziou P (2015). Faster title and abstract screening? Evaluating Abstrackr, a semi-automated online screening program for systematic reviewers. Syst Rev.

[9] Wallace BC, Trikalinos TA, Lau J, Brodley C, Schmid CH (2010). Semi-automated screening of biomedical citations for systematic reviews. BMC Bioinformatics.

[10] Lin JS, Evans CV, Johnson E, Redmond N, Burda BU, Coppola EL, Smith N (2018). Nontraditonal risk factors in cardiovascular disease risk assessment: a systematic evidence report for the U.S. Preventive Services Task Force.

